# A clinical prostate biopsy dataset with undetected cancer

**DOI:** 10.1038/s41597-025-04758-7

**Published:** 2025-03-11

**Authors:** Eduard Chelebian, Christophe Avenel, Helena Järemo, Pernilla Andersson, Carolina Wählby, Anders Bergh

**Affiliations:** 1 Department of Information Technology and SciLifeLab, Uppsala University, 752 37 Uppsala, Sweden; 2 Department of Medical Biosciences, Pathology, Umeå University, 901 85 Umeå, Sweden

**Keywords:** Prostate cancer, Computational biology and bioinformatics

## Abstract

Prostate cancer is a heterogeneous disease showing variability both among individuals and within a patient. While most cases are indolent, aggressive tumors require early intervention. Accurately predicting tumor behavior is challenging, contributing to overdiagnosis but also undertreatment. Current imaging methods may miss the most malignant areas, leading to biopsies often capturing non-malignant prostate tissue even if cancer is present elsewhere in the organ. This non-malignant tissue, however, holds potential as a source for novel diagnostic and prognostic markers. Our clinical dataset comprises men with raised prostate-specific antigen but whose initial prostate needle biopsies only contained benign tissue. Half of the paired patients remained cancer-free for over eight years, while the others were diagnosed with prostate cancer within 30 months of follow-up. We share these initial benign biopsies to enable the exploration of morphological changes in non-malignant tissue and the potential for improved diagnostic accuracy in the early identification of patients with prostate cancer.

## Background & Summary

Prostate cancer (PCa) is one of the most prevalent cancers globally, constituting approximately 7.3% of all cancer cases and 3.8% of all cancer-related deaths, with notable variations across different geographical regions^1^. PCa is characterized by its multifocal and heterogeneous nature, where patients may harbor multiple distinct tumor foci simultaneously, many of which are indolent and not clinically significant^2^. Presently, the primary approach for early PCa detection relies on the measurement of the prostate-specific antigen (PSA) in blood samples. Raised values ( > 3 ng/ml) suggest prostate pathology, though not necessarily cancer. The higher the PSA the higher the risk that a clinically significant PCa is present^3^. While the introduction of the PSA test led to higher detection rates of PCa, it has also resulted in a surge in overdiagnosis and overtreatment, given the high prevalence of patients with indolent prostate tumors who do not require treatment^4^.

Patients presenting elevated PSA levels generally undergo prostate magnetic resonance imaging (MRI) followed by MRI-guided and/or systematic needle biopsies^5,6^. Biopsies are stained employing hematoxylin-eosin (H&E) staining and examined by microscopy^7^. The International Society of Urological Pathology (ISUP) introduced the ISUP grading system in 2019, building upon the classical microscopy-based Gleason grading system and comprising five distinct grading groups^8^. ISUP grade 1 denotes a low risk of disease progression and where active treatment is unnecessary^9^. While ISUP grades 2 and 3 indicate intermediate risk, ISUP3 is more aggressive than ISUP2. Grades 4 and 5 are the most malignant PCa variants. Prostate needle biopsies typically involve the collection of approximately 12 samples taken from different predetermined locations and MRI-detected lesions. Given the limited size of needle biopsies, sampling less than 0.1% of the prostate volume, there is a risk that clinically significant tumor areas are missed^10^. Consequently, a substantial portion of these initial biopsies result in benign diagnoses, highlighting the need for improved detection methods^11^. Previous studies have indicated that cancers, to grow and spread, need to influence the benign parts of the tumor-bearing organ, and the nature and magnitude of these modifications are related to tumor aggressiveness^12^. Understanding such changes could provide valuable insights for early diagnosis and detection.

Currently, available prostate datasets primarily focus on comprehensive collections with cancer-containing biopsies designed for cancer detection purposes. In this category, the dataset accompanying the Prostate cANcer graDe Assessment (PANDA) Challenge^13^ stands out as the largest, including over 10,000 whole-slide images (WSI) biopsies from six different institutions to facilitate the development of a Gleason grading algorithm. Additional efforts have aimed to emulate the common scenario of having benign biopsies with undetected PCa by sampling benign cores in the proximity of detected cancer biopsies^14^. While this approach allows the analysis of changes in cancer-associated benign tissue, it may be considered too proximal to the lesion compared to the conditions encountered in routine clinical practice. Complementing these approaches, studies such as the DOCUMENT trial have highlighted the utility of epigenetic assays in identifying missed cancers in initial negative biopsies, emphasizing the potential for systematic integration of such tools in improving cancer detection in follow-up biopsies^15^. Thus, there is potential value in exploring biopsies classified as benign but where later follow-up and re-biopsies revealed that clinically significant cancer was present and presumably missed by the initial biopsies.

**Fig. 1:**
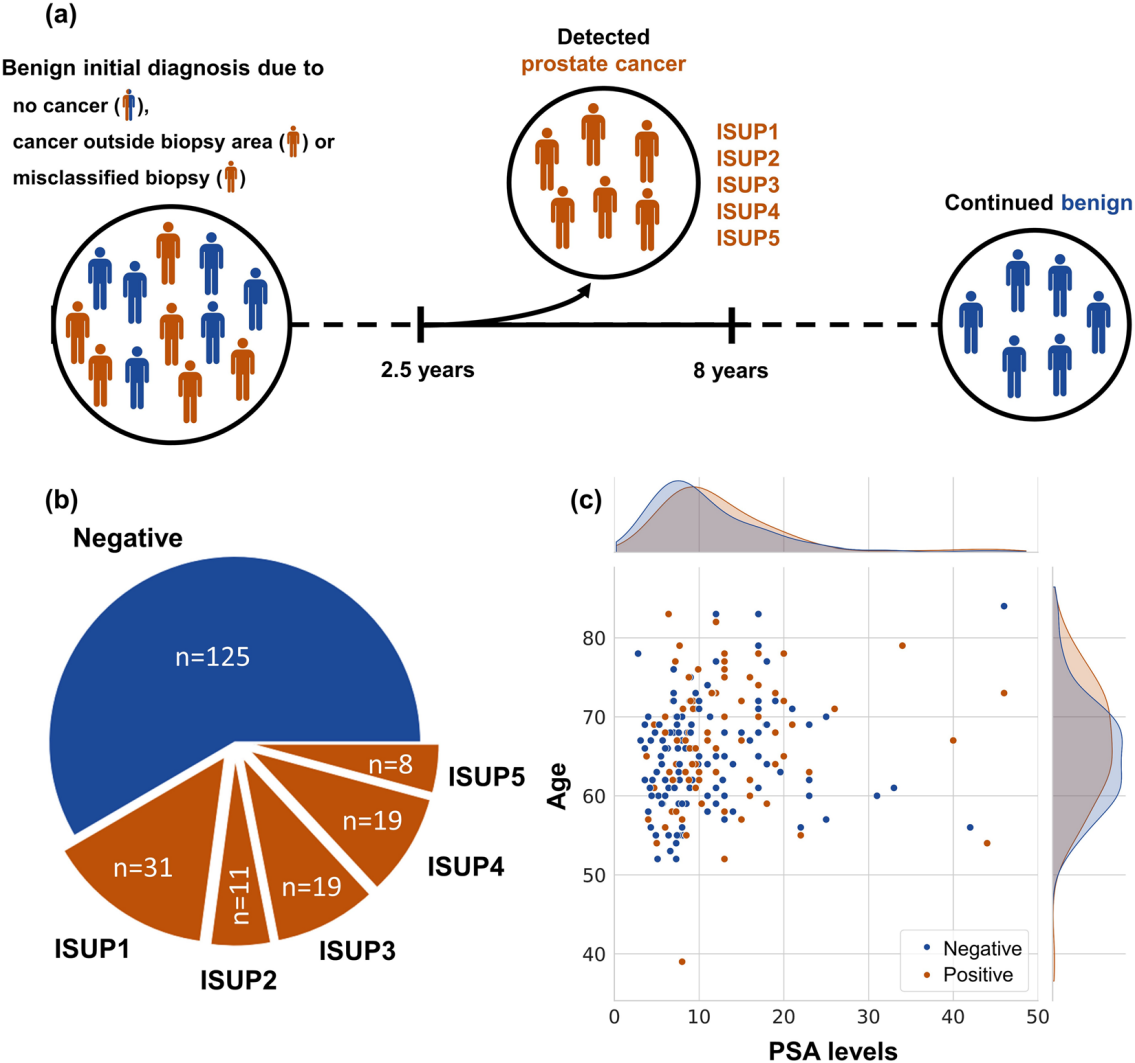
Summary of the dataset. **(a)** Data collection protocol. **(b)** Distribution of the patients. **(c)** The cohort was selected to have similar PSA and age distribution of negative and positive patients.

To address this, we introduce a novel dataset comprising digitized benign prostate H&E biopsies from men with raised PSA. The biopsies were systematically taken from different locations in the prostate and were not guided by MRI as, given the long follow-up times, the patients preceded the extended use of MRI for guiding biopsies. The dataset is uniquely designed, including paired samples from patients with comparable age and PSA levels, all initially diagnosed as benign but with different outcomes upon subsequent follow-ups and re-biopsies. While some patients remained cancer-free during at least eight years of follow-up, others were diagnosed with PCa within the subsequent 30 months of follow-up. The final processed dataset encompasses 213 patients, resulting in a total of 587 H&E prostate needle biopsies. Among these, 125 control patients with 333 biopsies exhibited no cancer development in at least eight years following the initial diagnosis. Conversely, 88 case patients with 254 biopsies were diagnosed with PCa of various ISUP grades within the 30 months following the initial diagnosis. Each case patient is accompanied by up to three control patients, paired for similar age, PSA and year of diagnosis, ensuring a robust comparative analysis. In Fig. 1, we provide a schematic view of the dataset and propose three reasons why an initial biopsy could appear benign: (1) no cancer was present initially, even for cases later diagnosed; (2) cancer was present but missed due to sampling outside the affected area; or (3) cancer was misclassified, though this is unlikely in our dataset, it can be still worth considering.

In this work, we present the dataset used in a prior clinically oriented work^16^. We envision the application of this dataset for uncovering new insights within the initially benign biopsies. Our scope goes beyond traditional diagnosis, aiming to study the long-range morphological effects induced by PCa in the benign parts of the organ. The dataset offers a unique opportunity to understand PCa progression, enabling the identification of potential markers for early detection by leveraging current computational methods. This, in turn, may open avenues for new diagnostic and therapeutic strategies.

## Methods

### Patient selection

Patients included in the dataset were retrospectively selected from records at the University Hospital of Umeå (*Norrlands universitetssjukhus*) in Umeå, Sweden between the years 1997 and 2016. The initial step involved identifying patients who had undergone a non-initial positive biopsy. Among this subset, individuals with an initial negative biopsy within the 30 months prior to the detection of PCa were selected. For each patient meeting these criteria, up to three control patients were selected based on similar PSA levels, age, and year of diagnosis. Control patients were required to have had initial negative biopsies and to have continued negative at re-biopsies for at least eight years of follow-up. Note that MRI-guided biopsies were only introduced in the region in 2015 so, due to the long follow-up times needed for this setup, all the patients underwent systematic biopsies. To comply with ethical standards and ensure patient confidentiality, all data was anonymized upon collection. Ethical approval for use and publication of the data was granted by the *Regionala Etikprövningsnämnden* in Umeå, DNR 2010/366-31M. The data remains fully anonymized and does not allow for identification of any individuals.

In the process of patient selection, clinical data was also acquired from the registries at the Department of Pathology at the University Hospital of Umeå. Apart from the selection parameters such as PSA levels and age, a key factor of our dataset is the ISUP grade assigned to patients upon the late discovery of PCa. Less aggressive PCa lesions, classified as ISUP1, are generally associated with fewer cancer-related alterations in adjacent tissues^9,12^. Additionally, ISUP1 lesions can exhibit morphological characteristics that resemble benign tissue, thus potentially posing a challenge in the initial diagnosis of the biopsies. The unique aspect of our dataset lies in the identification of patients initially diagnosed as benign whose subsequent re-biopsies revealed clinically significant cancer (ISUP2–5) within the 30 following months. Studying the morphological nuances between them is fundamental to understanding the progression dynamics of PCa.

### Digitization

The biopsy specimens underwent digitization using the 3D Histech Pannoramic 250 scanner (3D HISTECH Ltd., Budapest, Hungary), operating at a magnification of 20 × with a pixel size of 0.2428 *μ*m/pixel. The process yielded MRXS files in .mrxs format, including eight levels of pyramidal downsampling. The pyramidal format enables efficient data storage and manipulation in subsequent computational analyses.

### Preprocessing

To ensure the technical viability of the dataset, we implemented the preprocessing workflow proposed by Lu *et al*.^17^ The preprocessing consists of WSI tissue detection and segmentation modules, a fundamental first step in digital pathology. This generates tissue masks, enabling the identification and exclusion of biopsies with insufficient tissue or noticeable artifacts.

### Statistical analysis

In order to confirm the effectiveness of the patient pairing, multivariate logistic regression analysis was conducted to explore the relationship between patient age and PSA at the time of benign diagnosis with the future development of PCa. We explored the R-squared (*R*^2^) of the model as well as the individual p-values for both variables. Additionally, we calculated the area under the receiver operating characteristic curve (*AUC*) to establish a baseline.

## Data Records

The complete clinical prostate biopsy dataset, where the cancer was initially undetected, is openly accessible at the Swedish National Data Service^18^. This dataset includes 587 WSI H&E-stained prostate needle biopsies, originating from 213 patients initially diagnosed as benign.

Each slide in the dataset is provided in MRXS format, which includes a .mrxs file with the image and a folder containing .dat files. The naming convention for each slide follows the pattern *patient_NNN_SSS*. Here *NNN* represents the anonymous code for the patient and *SSS* indicates the specific biopsies within the slide. For instance, *patient_137* has associated four image files: *patient_137_ABC*, *patient_137_DEF*, *patient_137_GHK* and *patient_137_LMN* with three parallel slides each. On average, each patient has three associated slide files.

Each patient corresponds to a row in the description.csv file, as detailed in Table 1. For each patient, we collected the *PSA* and *Age* information at the time of the initial benign diagnosis, which we do not share for anonymization reasons. The *Positive* column indicates the presence of PCa within the 30 months of follow-up after benign diagnosis, denoted by a value of *1* (one) or *0* (zero) if subsequent diagnoses remain benign over at least the following eight years. In cases of positive diagnoses, the *ISUP* column reflects the ISUP grade of the late detected cancer, ranging from *0* (zero) to *1,2,3,4,5* (one, two, three, four, five) depending on the aggressiveness. Each positive patient was matched with up to three negative control patients for comparative analysis.

**Table 1:** Per slide information included alongside the images.

Column name	Explanation
Patient_N	Anonymous patient ID.
PSA	Prostate-specific antigen value at the time of biopsy collection.
Age	Age range of the patient at the time of biopsy collection.
Positive	Cancer status in the subsequent 30 months of follow-up after biopsy collection.
ISUP	ISUP grade of the cancer found in the subsequent 30 months of follow-up after biopsy collection.

The data is packaged into a undetected_prostate_cancer.zip ZIP file. The ZIP file is split into 50 GB parts for convenience.

## Technical Validation

The dataset was acquired during routine clinical practice. Upon initial diagnosis, all collected patients underwent evaluation by a histopathologist, establishing a benign diagnosis. Subsequent evaluations by histopathologists upon re-biopsies were conducted to determine the absence or presence of cancer and, in the latter case, to assign a grade. Therefore, the dataset establishes a link between between initial benign diagnoses and potential subsequent cancer outcomes. The multiple follow-up evaluations contribute to the accuracy and reliability of the data.

The clinical variables were collected in a paired manner, ensuring that patients, regardless of their eventual cancer status, are not anticipated to exhibit significantly different years of diagnosis, age, or PSA levels at the time of the first negative biopsy. As depicted in Fig. 1c, which illustrates the distribution of age and PSA levels for both negative and positive cases, the clinical variables alone are insufficient to distinguish between the two groups. These observations were confirmed by the multivariate logistic regression, yielding *R*^2^ = 0.022 and p-values for both independent variables well above the conventional significance level of 0.05. Finally, the *AUC* = 0.59 was only slightly above random chance. This highlights the necessity for more advanced computational methods to identify potential changes present at the initial diagnosis that could be indicative of later cancer diagnosis.

**Fig. 2:**
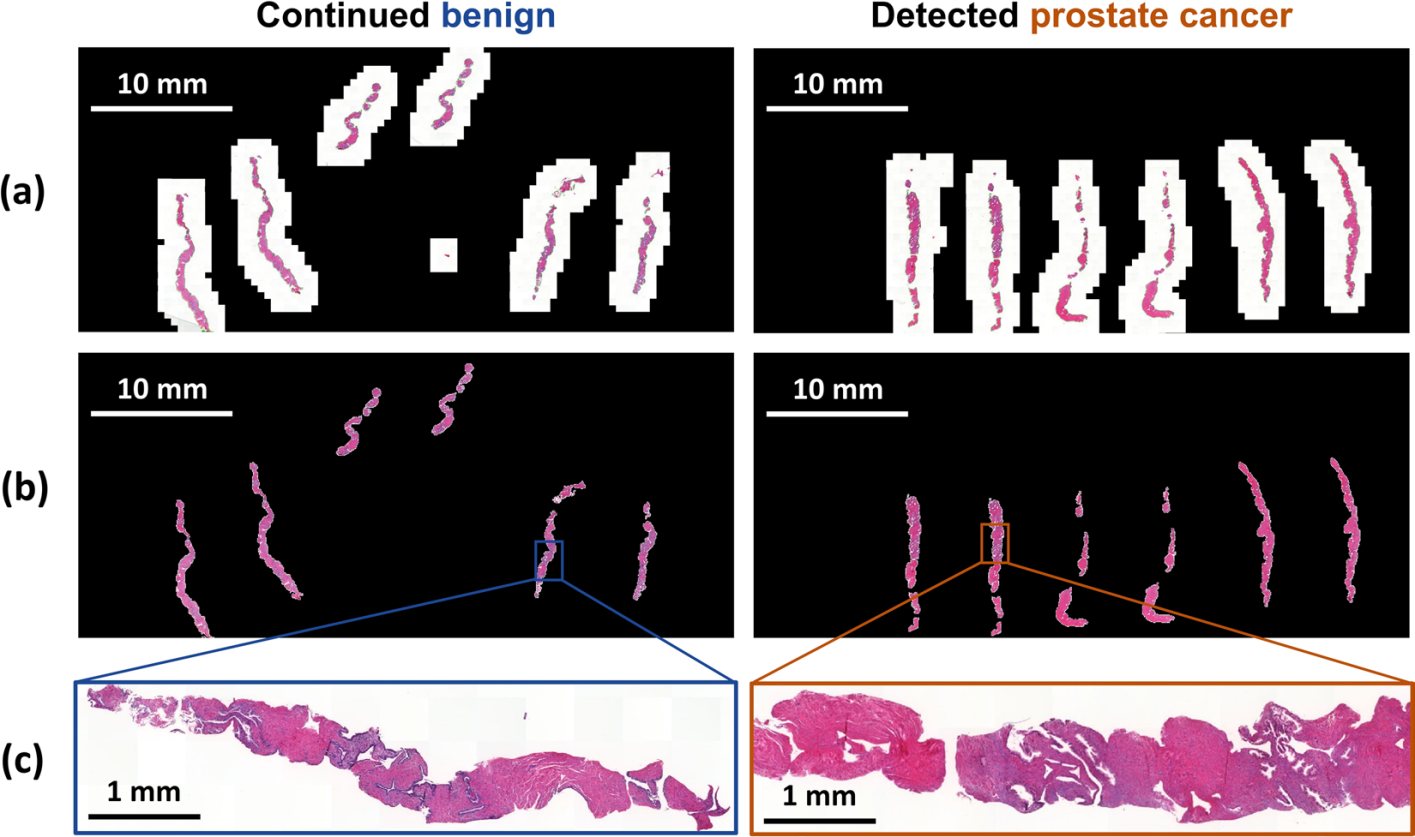
Example data for one of the slides from a patient that continued being benign or had late detection of prostate cancer. **(a)** Original slide and **(b)** stitched detected tissue^17^. **(c)** Zoom in of a region.

To assess the suitability of the dataset with deep learning workflows, we conducted initial preprocessing steps for tissue detection and segmentation. Figure 2b presents the detected tissue for two examples of slides, one from a patient whose condition remained benign and another from a patient later diagnosed with PCa. This analysis facilitated the identification and elimination of slides with corrupted files or undetected tissue. Notably, no further patients were excluded, only specific samples were excluded based on the preprocessing outcome.

## Usage Notes

MRXS files can be conveniently inspected using compatible viewer software, such as QuPath^19^, ImageJ^20^ or TissUUmaps 3^21^. We have provided example data from TissUUmaps in Fig. 2 and an interactive view accessible at https://undetected-prostate-cancer.serve.scilifelab.se/.

The data is readily available to be used in deep learning frameworks. The software utilized for WSI tissue detection and segmentation^17^ also includes tiling and feature extraction modules that facilitate image classification. Downsampled versions of the images enable access to lower levels of the resolution pyramid depending on the application. The dataset was previously explored in a weakly-supervised learning framework^16^.

## Data Availability

MRXS files were anonymized using the module in https://github.com/bgilbert/anonymize-slide. The WSI segmentation module proposed in CLAM^17^ was used for preprocessing the slides https://github.com/mahmoodlab/CLAM. The images were inspected using TissUUmaps https://tissuumaps.github.io/.
